# Supplementary figures and images for: Comparative transcriptomics reveals the difference in early endosperm development between maize with different amylose contents
Source: PeerJ. 2019 Aug 28;7:e7528. doi: 10.7717/peerj.7528 (PMC6717500; doi:10.7717/peerj.7528)

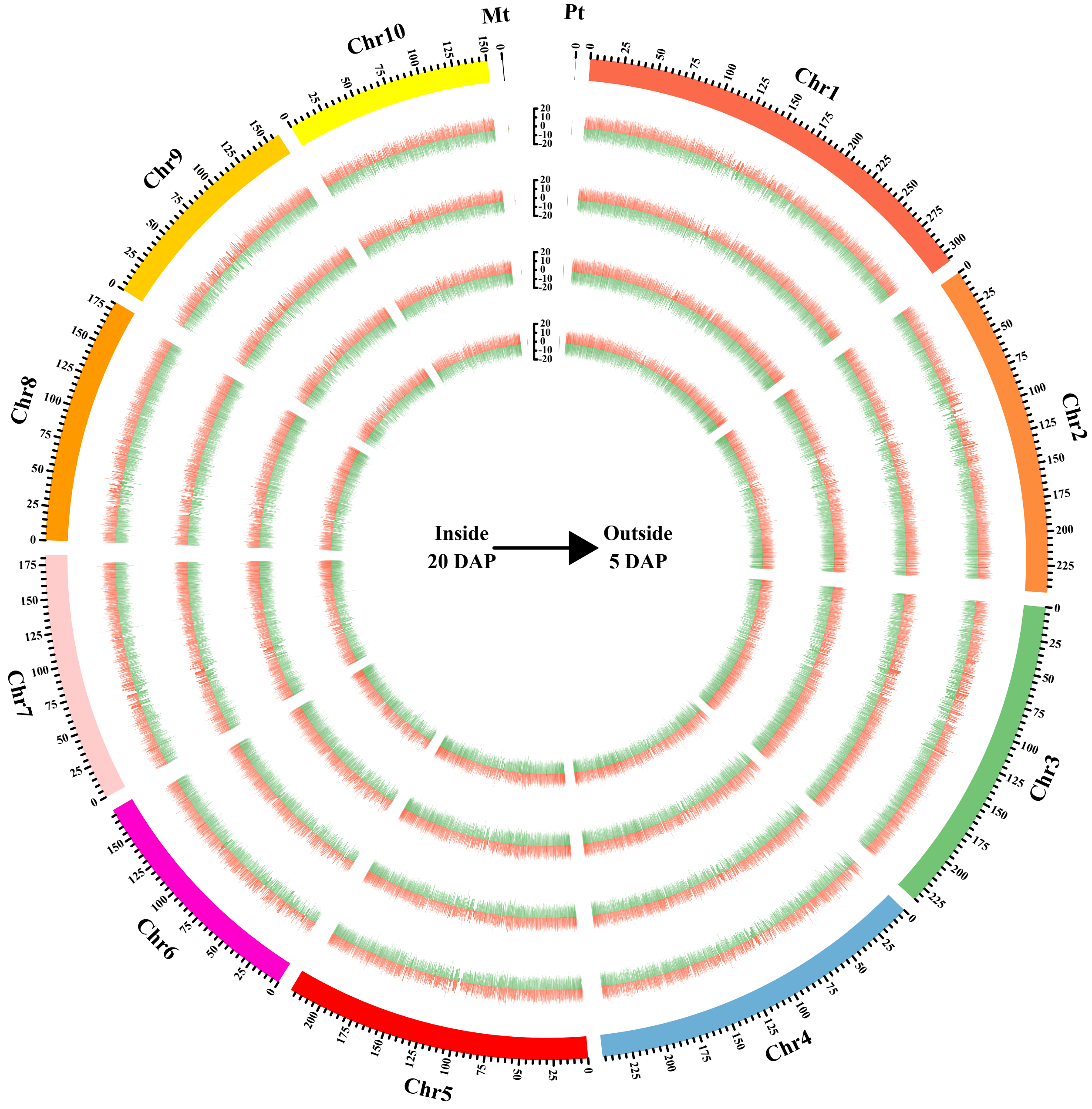

Supplement: Figure S1 — The reads at 5, 10, 15 and 20 DAP mapped to maize reference genome sequences are shown from the outside to the inside of the Circos image. The red and green colours represent the normalized read numbers of SD609 and HS68, respectively. [file peerj-07-7528-s001.pdf]

A

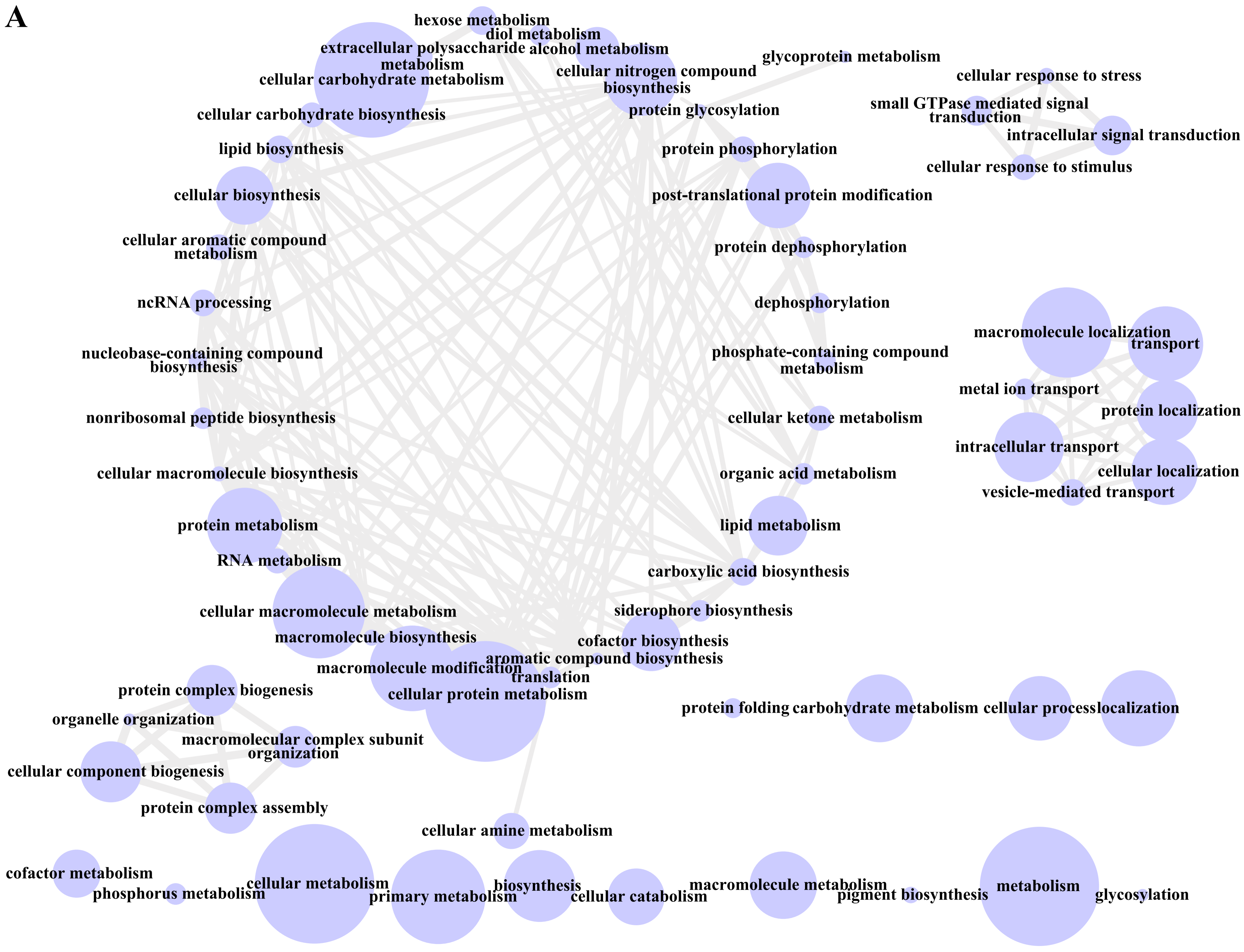

B

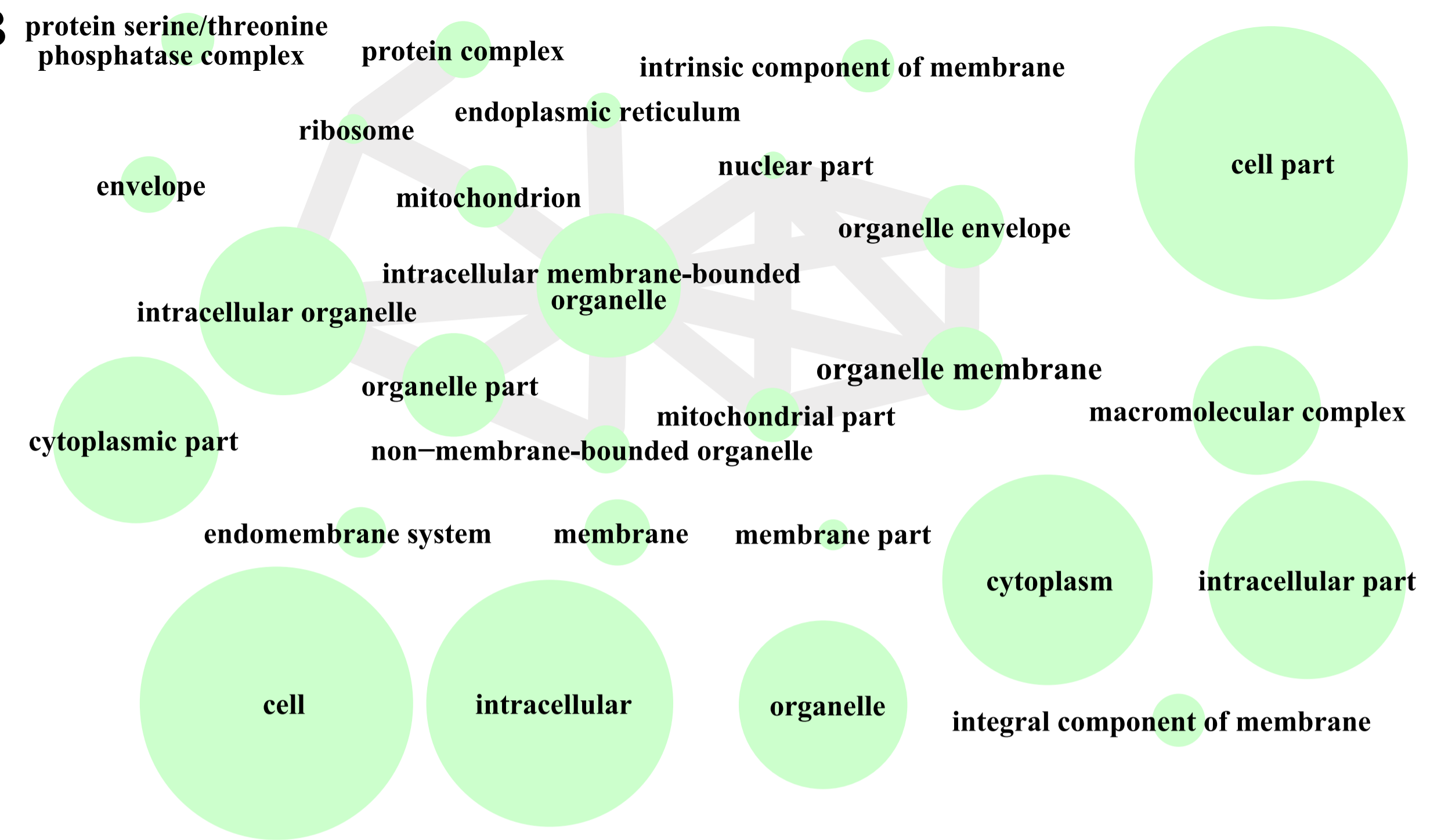

C

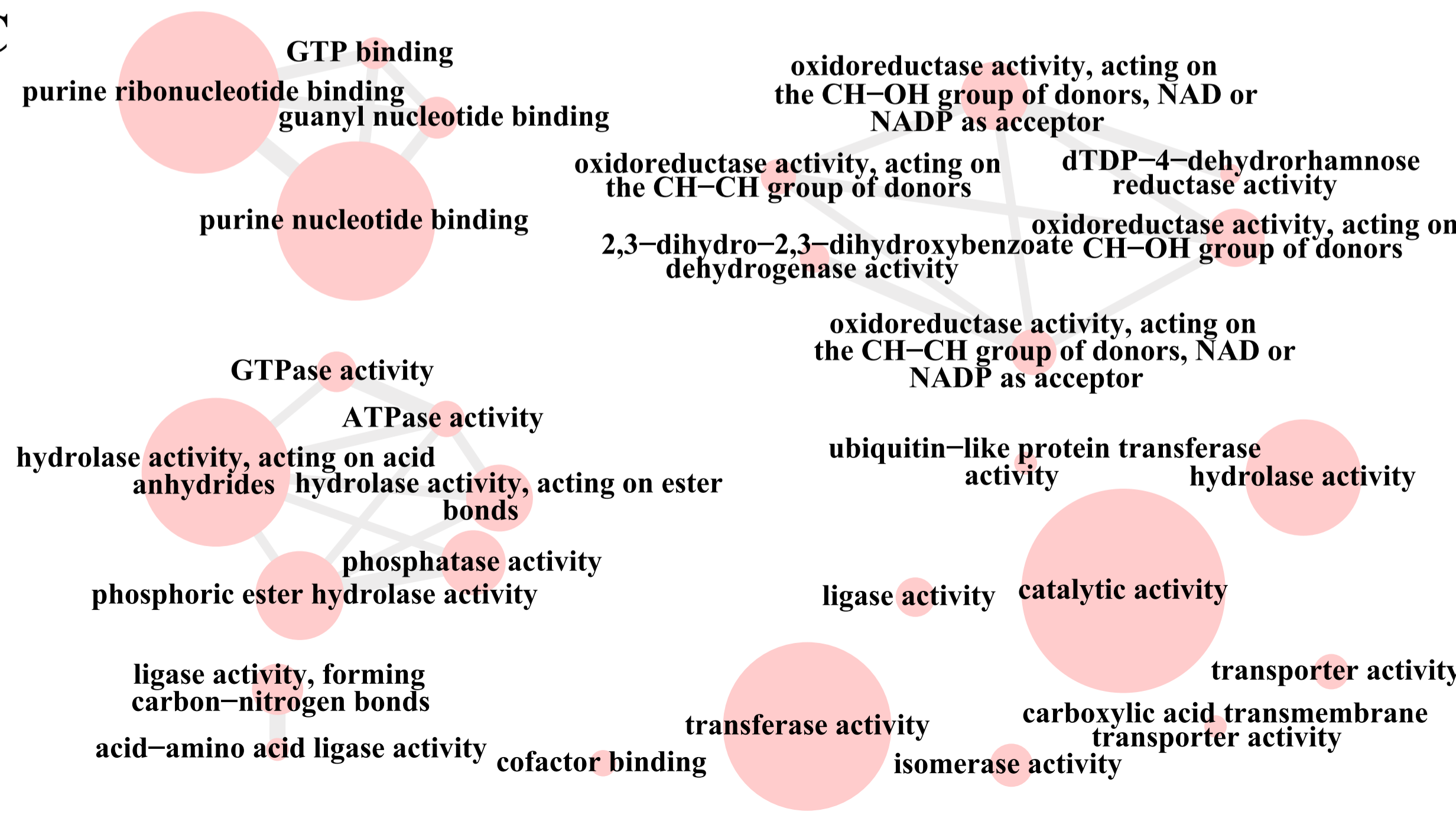

Supplement: Figure S2 — (A) The enriched GO terms in the “biological process”, (B) “cellular component”, and (C) “molecular function” categories are shown. [file peerj-07-7528-s002.pdf]

# DEGs

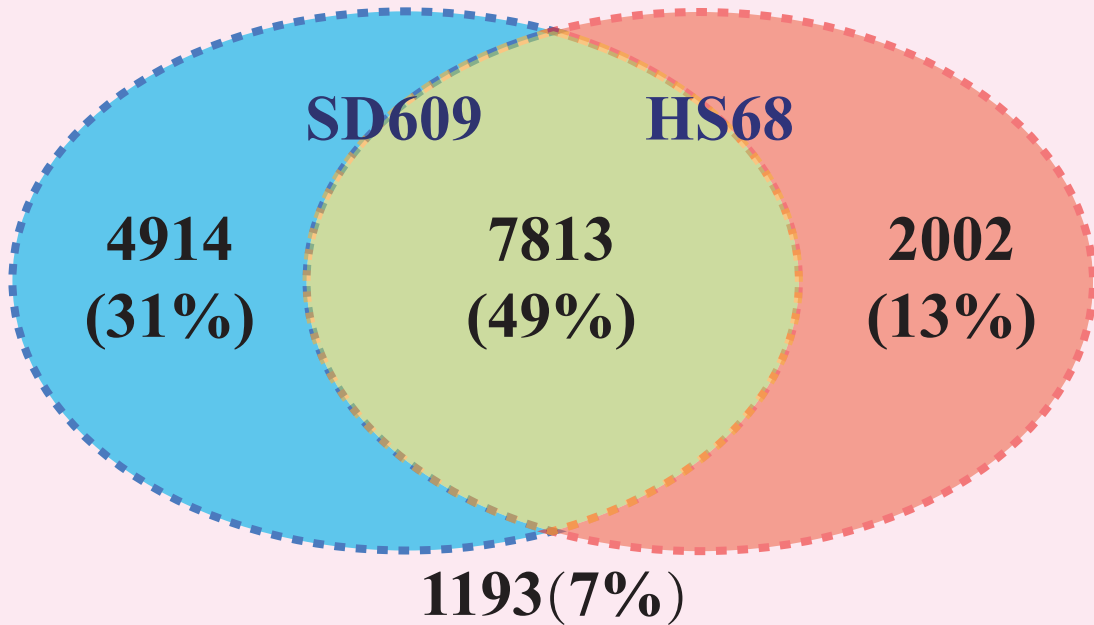

Supplement: Figure S3 — A total of 1,193 DEGs were differentially expressed between SD609 and HS68, 7,813 DEGs were consistently expressed in SD609 and HS68 at the different development stages, and 4,914 and 2,002 DEGs were specifically detected in SD609 and HS68, respectively. [file peerj-07-7528-s003.pdf]

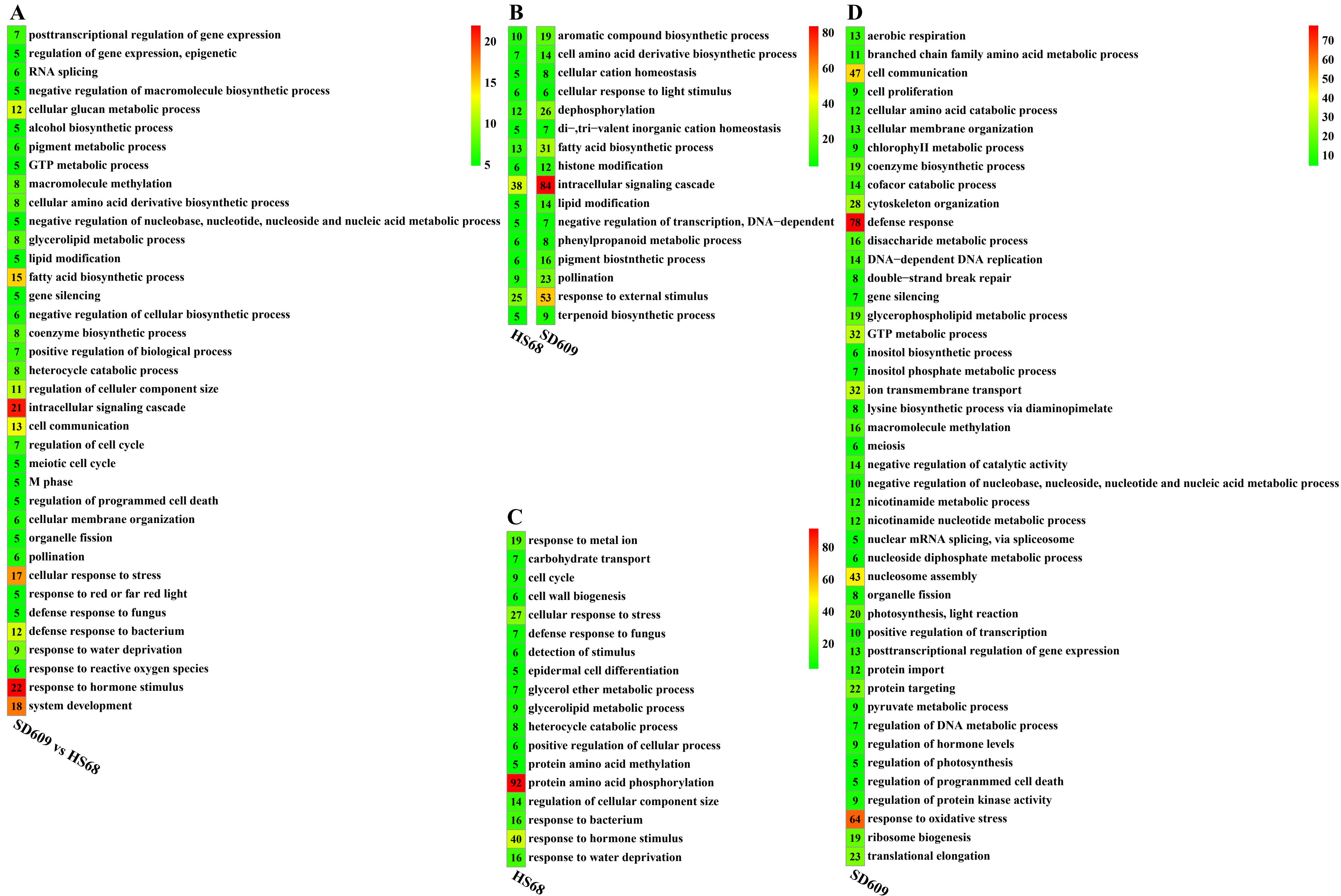

Supplement: Figure S4 — (A) Functional annotation of 1,193 DEGs between SD609 and HS68. (B) Shared biological processes regulated by the material-specific DEGs of SD609 and HS68. (C) Specific biological processes of HS68 that are regulated by the material-specific DEGs of SD609 and HS68. (D) Specific biological processes of SD609 that are regulated by the material-specific DEGs of SD609 and HS68. Only biological processes with P values < 0.0001 and FDR < 0.01 are shown. [file peerj-07-7528-s004.pdf]

**A**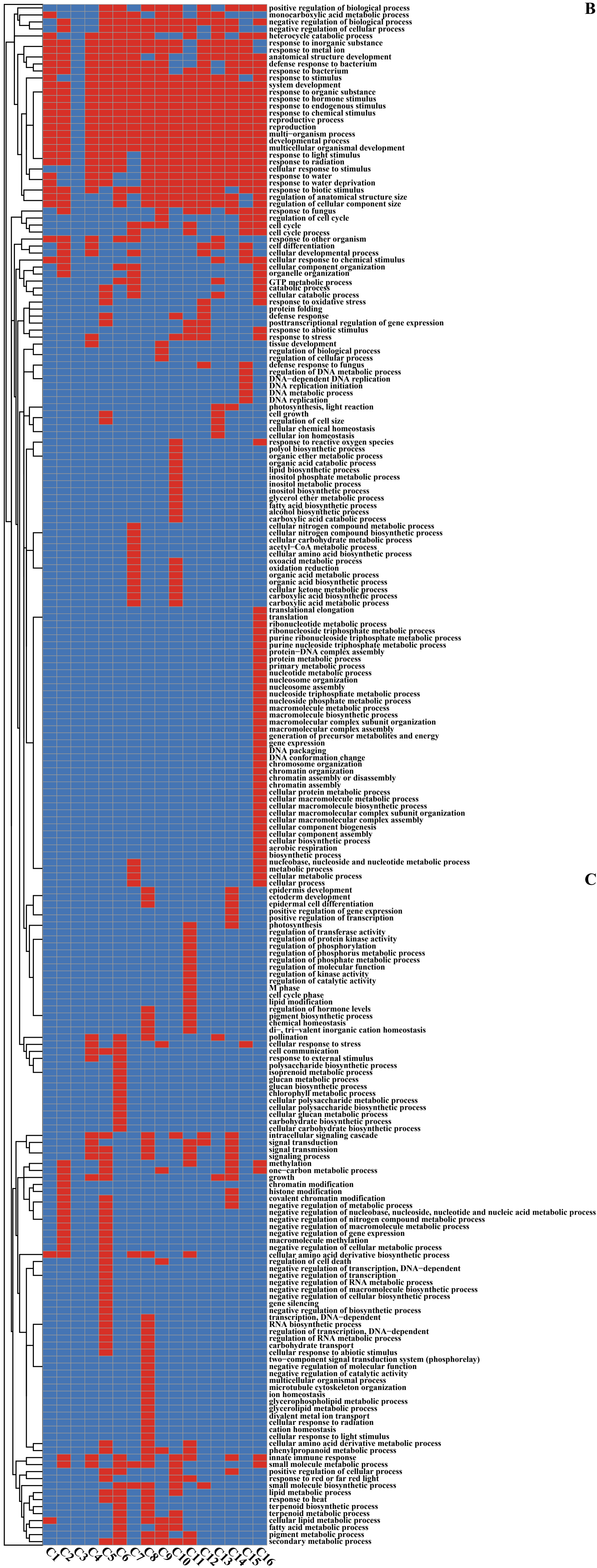**B**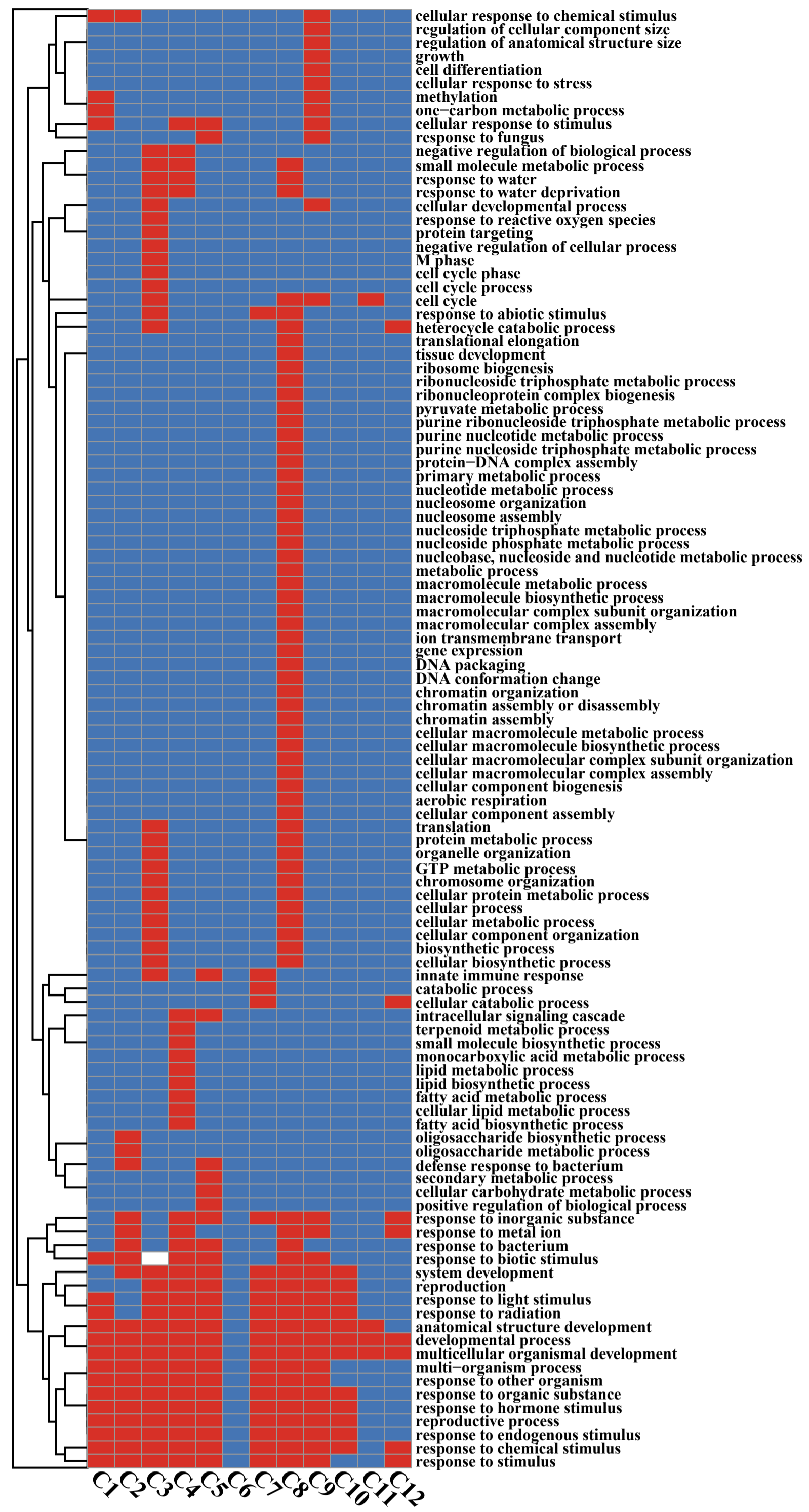**C**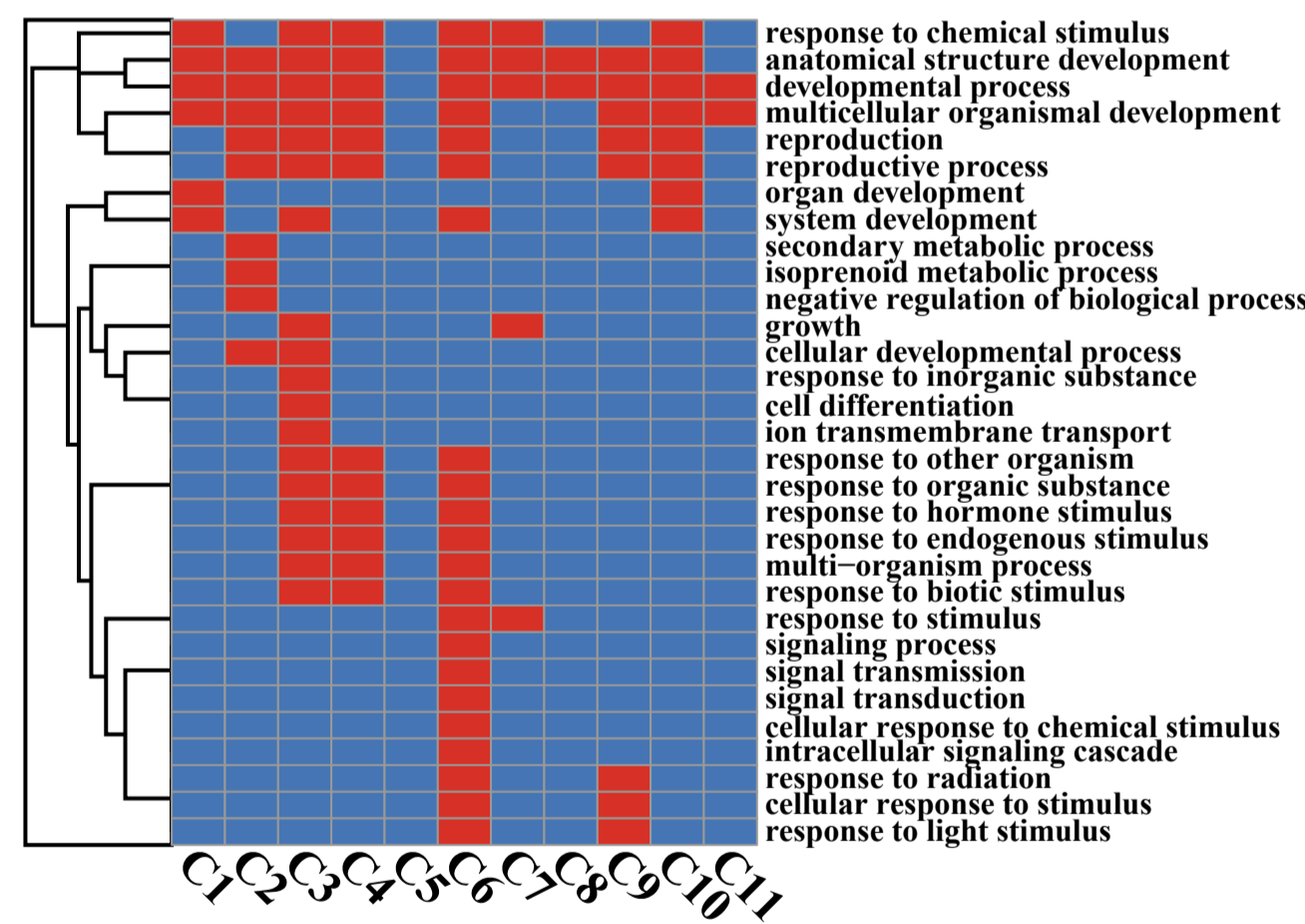**■ Annotation****■ No annotation**

Supplement: Figure S5 — Only biological processes with a P value < 0.0001 and FDR < 0.01 are shown. (B) Functional analysis of coexpression clusters of SD609. (C) Functional analysis of coexpression clusters of HS68. (A) Functional analysis of shared DEGs in the coexpression clusters between SD609 and HS68. [file peerj-07-7528-s005.pdf]

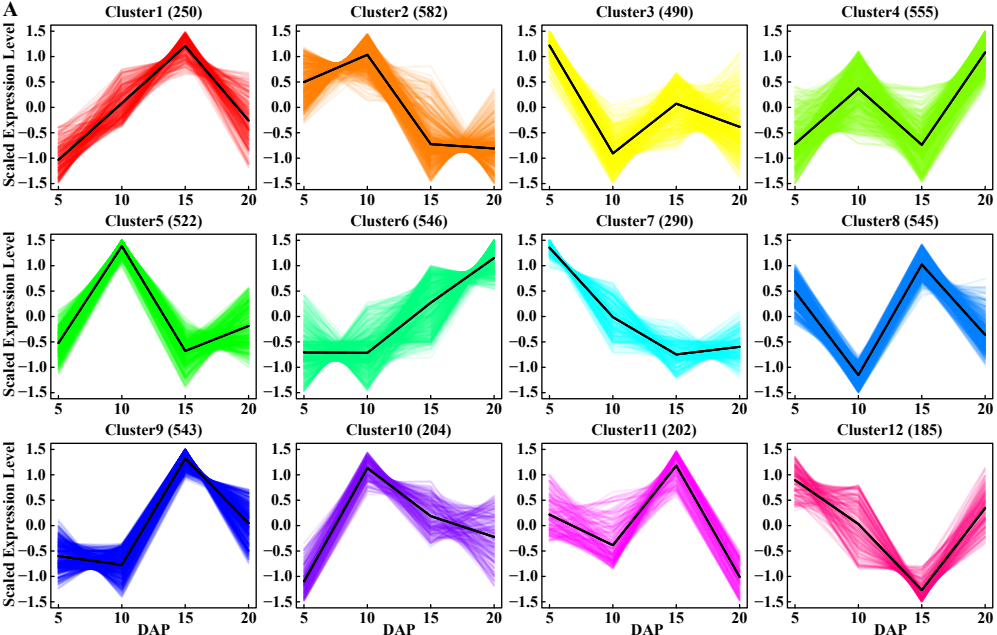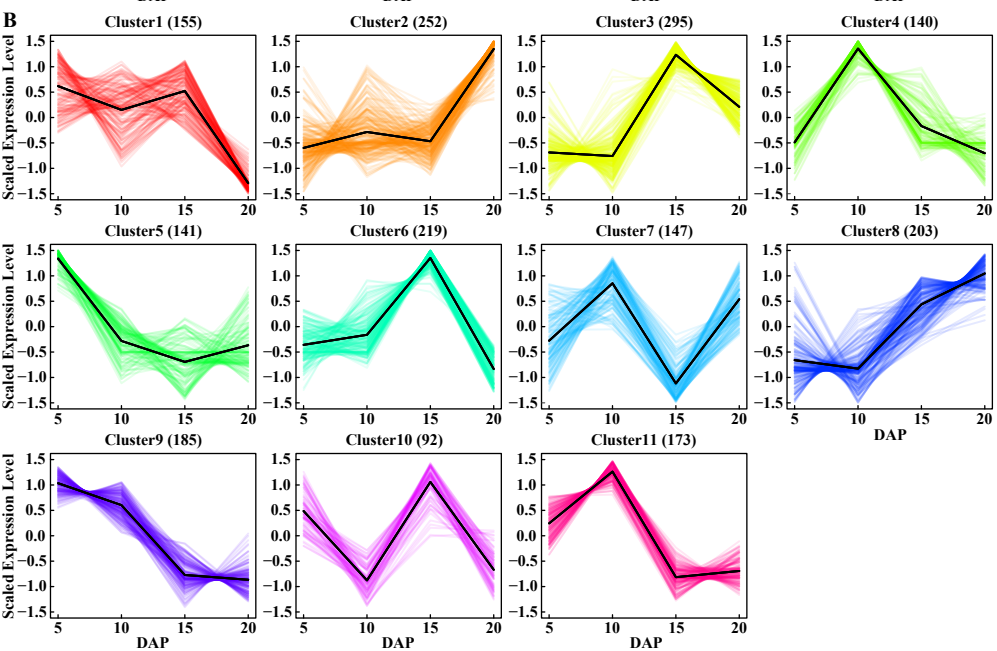

Supplement: Figure S6 — (A) Twelve clusters were characterized by the fluctuating expression of gene sets at 5, 10, 15 and 20 DAP in SD609. (B) Eleven clusters were characterized by the fluctuating expression of gene sets at 5, 10, 15 and 20 DAP in HS68. The up- and downregulated gene sets are staggered or depicted consecutively during the development of the maize endosperm. The scaled expression levels of the DEGs are provided on the y-axis, the developmental stages are shown on the x-axis, the coloured lines represent the individual gene expression clusters, and the trend in the expression of each gene set is depicted by a black line. “n” represents the number of DEGs. [file peerj-07-7528-s006.pdf]

## Relation between module eigengenes

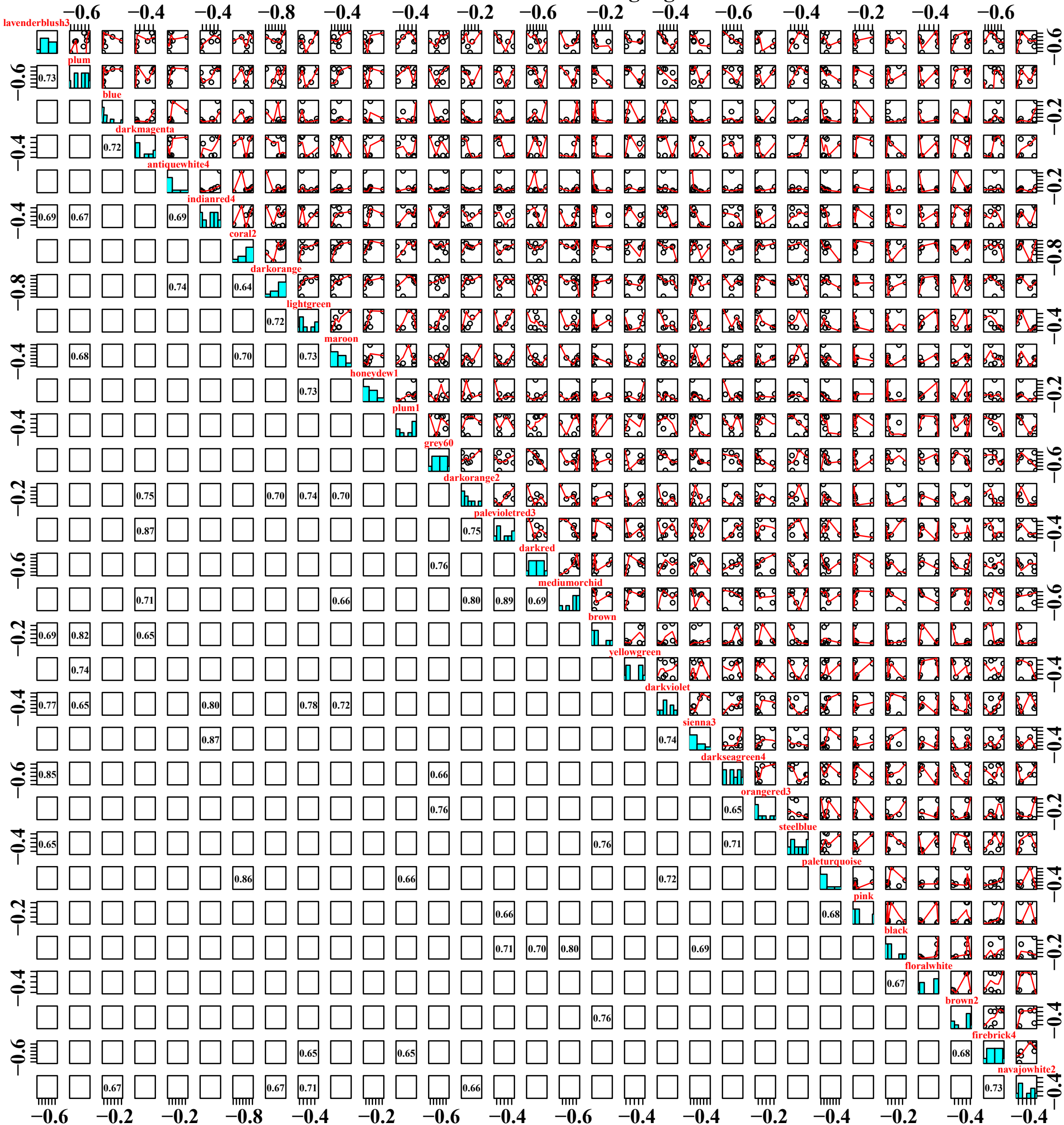

Supplement: Figure S7 — The diagonals show the distribution. The lower left section shows a bivariate scatterplot with a fitting line, and the upper right section shows the correlation coefficient and the significance level. [file peerj-07-7528-s007.pdf]
